# Pulmonary mucoepidermoid carcinoma in the Chinese population: A clinical characteristic and prognostic analysis

**DOI:** 10.3389/fonc.2022.916906

**Published:** 2022-08-24

**Authors:** Qiuyu Li, Xuejing Wei, Yanfei Wang, Chang Liu, Boshi Fan, Cheng Lv, Wenzhe Si, Min Li

**Affiliations:** ^1^ Department of Respiratory and Critical Care Medicine, Peking University Third Hospital, Beijing, China; ^2^ Department of Pathology, Beijing Friendship Hospital, Capital Medical University, Beijing, China; ^3^ Peking University Cancer Hospital and Beijing Cancer Institute, Day Oncology Unit, Key Laboratory of Malignant Tumor Pathogenesis and Transformation Research, Ministry of Education, Beijing, China; ^4^ Department of Emergency, Beijing Friendship Hospital Affiliated to Capital Medical University, Beijing, China; ^5^ Department of Thoracic Surgery, the Sixth Medical Center of Chinese PLA General Hospital, Beijing, China; ^6^ Department of Clinical Laboratory, Peking University Third Hospital, Beijing, China; ^7^ Department of Pathology, Peking University Third Hospital, Beijing, China

**Keywords:** pulmonary mucoepidermoid carcinoma, clinical features, chinese population, prognosis, pathological features

## Abstract

**Background:**

Mucoepidermoid carcinoma is dominant in salivary glands and rarely occurs in the lung. Primary pulmonary mucoepidermoid carcinoma is a type of non-small-cell lung cancer, but the prognostic factors in Chinese patients remain controversial. This investigation aimed to review cases of pulmonary mucoepidermoid carcinoma, analyse the prognosis of this disease.

**Methods:**

Patients with pathologically proven pulmonary mucoepidermoid carcinoma were screened at the Department of Respiratory and Critical Care Medicine at the Peking University Third Hospital, Beijing Friendship Hospital Affiliated to Capital Medical University, and Peking University Cancer Hospital for inclusion in this retrospective study. Demographic data, including age, sex, clinical symptoms, smoking, alcohol consumption, allergies, family history, imaging findings, fibrobronchoscopy findings, surgical procedures, tumour location and pathologic stage, were collected. Telephone follow-up was conducted for all patients not lost to follow-up. The associations of sex, age, smoking, tumour differentiation, tumour size, lymph node metastasis, pathologic stage, and patient survival were retrospectively analysed. Kaplan–Meier, univariate and multivariate analysis curves were used to analyse patient prognosis and prognostic factors.

**Results:**

Thirty-one patients, comprising 23 males and 8 females, were enrolled in the analysis. The mean age was 60.77 ± 11.44 years. The first symptom was nonspecific, with cough being the most common (21/31, 67.77%); smokers accounted for 16 of the 31 patients, and ten patients had a history of alcohol consumption. Overall, the tumours could occur in either lobe of the lungs; tumours occurred in the right lung in 19/31 patients, and tumours occurred in the left lung in 12/31 patients. Regarding TNM stage, 10 patients had stage I (5 with stage 1a, 5 with stage 1b), 5 had stage II (1 with stage 2a, 4 with stage 2b), 3 had stage III (1 with stage 3a, 2 with stage 3b), and 13 had stage IV (10 with stage 4a, 3 with stage 4b). In our Cox univariate survival analysis of patients with pulmonary mucoepidermoid carcinoma, we found that TNM stage IV, degree of differentiation and lymph node metastasis were risk factors for pulmonary mucoepidermoid carcinoma and that degree of differentiation was an independent risk factor.

**Conclusion:**

The clinical, radiographical and pathological features of pulmonary mucoepidermoid carcinoma were systemically analysed and summarized, and the degree of differentiation and lymph node metastasis, as well as prognostic factors in addition to clinical stage, were confirmed.

## Introduction

Mucoepidermoid carcinoma (MEC) of the lung is one of the rarer lung malignancies, has no specific clinical manifestations and presents mostly as irritant symptoms of the bronchi, including cough, expectoration, haemoptysis, chest pain, chest tightness, and fever (1). Patients with tumours obstructing the trachea present mostly with atelectasis and inflammation. However, tumours that are small or grow around the lungs do not cause symptoms (2). The literature reports no clear association between smoking and this condition (3). Pathological diagnosis is the gold standard for the diagnosis of the disease, and in today’s era of precision medical treatment in particular, accurate pathological diagnosis is the top priority. The general appearance of MEC of the lung is mostly round or irregular. The size of the tumour is generally 3–5 cm, and the section is grey and white with brittle matter. Pulmonary MEC (PMEC) can be divided into two types, low-level and high-level, according to morphology and the cell score (4). Low-grade tumours have a predominantly cystic component with inconspicuous cellular atypia, and the tumours are mostly nonnecrotic with areas of calcification. High-grade tumours are less common and present with heteromorphic cells, karyorrhexis, necrosis and regional lymph node metastasis. The literature on the common pathological grade of PMEC is mixed. In one study, 49 of 69 MECs were low grade (5), whereas in another study involving 11 patients, 8 had high-grade disease and 3 had low-grade disease (6). The development of immunohistochemical techniques plays an irreplaceable role in pathological classification and differential diagnosis because of the complex composition of the disease; sometimes the biopsy results, the results of intraoperative frozen section and even those of postoperative paraffin pathology cannot accurately report the case classification, and immunohistochemistry is necessary to assist the diagnosis. TNM stage and tissue grade are independent risk factors for PMEC prognosis (7, 8). Turnbull et al. (9) showed that PMECs are generally considered low-grade tumours and have a significantly better long-term prognosis than non-small-cell lung cancer (NSCLC), which was supported by other studies (10). Studies have shown that the more advanced the stage is, the lower the patient survival time and the more aggressive the high-grade tumour histology will be. Patients with PMEC without any lymph node metastasis who undergo complete resection are expected to be cured (11). It has also been documented that age ≥50 years, a peribronchial growth pattern, tumour size ≥3 cm, and Ki-67 labelling index ≥ 10% are all poor prognostic factors for PMEC (12). Because of the relatively low incidence of this disease clinically, there have been few investigations on its prognosis. This investigation aimed to review cases of PMEC, analyse the prognosis of this disease.

## Methods

### Study population and design

This observational cross-sectional study was conducted in the Department of Respiratory and Critical Care Medicine at the Peking University Third Hospital, Beijing Friendship Hospital Affiliated to Capital Medical University, and Peking University Cancer Hospital. We enrolled all inpatients admitted to these hospitals from March 2016 to March 2022.

The inclusion criteria were as follows: (I) pathological diagnosis of PMEC and (II) age ≥18 years. The exclusion criteria were as follows: 1) length of hospitalization <1 day or 2) refusal to participate in this study.

The study protocol was approved by the Ethics Committee of Peking University Third Hospital, Beijing Friendship Hospital Affiliated with Capital Medical University, and Peking University Cancer Hospital (No. IRB00006761-M2022102, 2022-P2-137-01, 2020KT103). If a patient was admitted to the Department of Respiratory and Critical Care Medicine more than once, data were collected from only the first hospitalization.

### Data collection

Patient data, including age, sex, clinical symptoms, smoking, alcohol consumption, allergies, family history, imaging findings, fibrobronchoscopy findings, surgical procedures, tumour location and pathologic stage, were collected from medical records.

Telephone follow-up was conducted for all patients not lost to follow-up. The associations between sex, age, smoking, tumour differentiation, tumour size, lymph node metastasis, and pathologic stage and patient survival were analysed. The patients were followed up every three months after discharge to evaluate survival, recurrence, treatment changes, and treatment-related complications.

### Histologic review and grading

Diagnostic H&E slides were reviewed by two pathologists, and each carcinoma was graded according to the World Health Organization (WHO) classification of tumours of the lung, pleura, thymus and heart (13). MEC is divided into high-grade and low-grade carcinomas. The criteria for distinguishing high-grade and low-grade tumours are as follows: low-grade malignant tumours are mainly cystic components, have no obvious cell abnormity, have no necrotic areas in most cases, and have observable calcification. Heteromorphic cells, mitotic-phase cells, necrosis and regional lymph node metastasis are found in highly malignant tumours.

### Survival analysis

A Cox regression model was used to analyse the predictors of all-cause death and infection. Since the follow-up data may be affected by the baseline data, the baseline data and follow-up data were modelled. First, Kaplan–Meier analysis (for classified variables) or a univariate Cox regression model (for continuous variables) were used to screen the variables that may be independent predictors. Only variables with P values less than 0.1 in the Kaplan–Meier analysis or univariate Cox regression were included in the final multivariate Cox regression model. The potential predictors of all-cause death included age, sex, pathological grade, lymph node metastasis and tumour stage. The corresponding segmental time-related variables were established according to whether the patient died during the specific follow-up time. The results were expressed as the relative risk (HR) and 95% confidence intervals (CI). A p value less than 0.05 was considered to indicate a significant difference. SPSS 10.0 was used for statistical analysis.

### Statistical analysis

Data were analysed using SPSS 23.0 software (IBM, New York, USA). Continuous variables are expressed as the means ± standard deviations. Categorical variables are expressed as frequencies and proportions. One-way ANOVA was used to evaluate differences among means across categories of nutritional risk. The chi-squared test or Fisher’s exact test was used for categorical variables.

## Results

### Demographic and clinical characteristics of the participants

During the study period, 40 patients with pathologically proven PMEC were admitted to the participating hospitals (including 7 patients admitted to Peking University Third Hospital, 16 patients admitted to Peking University Cancer Hospital, and 8 patients admitted to Beijing Friendship Hospital Affiliated to Capital Medical University). Nine patients were excluded due to missing data. Ultimately, 31 patients were enrolled in the analysis, consisting of 23 males and 8 females. The mean age was 60.77 ± 11.44 years, and the patients’ ages ranged from 31 to 77 years.

For these patients, the first symptom was nonspecific, with cough being the most common (21/31, 67.77%). None of the patients had a previous history of neoplasia; 4/31 of the patients had a family history of tumours, and 1/31 had a family history of lung cancer. There were more male patients, with a male-to-female ratio of 2.875:1 (23/8). Smokers accounted for 16/31 of the total population. Ten patients had a history of alcohol consumption, and all of them were male. Only one male patient had a previous history of allergies. The clinical data of all patients are detailed in **Table 1**.

**Table 1:** Clinical and follow-up data of 31 patients with pulmonary mucoepidermoid carcinoma.

Number	Total (n=31)	Low grade (n=25)	High grade (n=6)	P value
Sex (male/female)	23/8	20/5	3/3	0.132
Age	60.77 ± 11.44	60.280 ± 12.337	62.833 ± 6.998	0.632
Location				
upper left lung	9 (29%)	7 (28%)	2 (33.3%)	0.382
lower left lung	3 (9.6%)	3 (12%)	0 (0%)	
right-sided pleura	1 (3.2%)	1 (4%)	0 (0%)	
upper right lung	7 (22.6%)	4 (16%)	3 (50%)	
lower right lung	7 (22.6%)	7 (28%)	0 (0%)	
middle right lung	5 (16.1%)	4 (16%)	1 (16.7%)	
Symptoms				
cough	21 (67.7%)	18 (72%)	3 (50%)	0.301
bloody sputum	9 (29%)	6 (66.7%)	3 (50%)	0.208
haemoptysis	5 (16.1%)	4 (16%)	1 (16.7%)	0.968
chest tightness	5 (16.1)	5 (20%)	0 (0%)	0.232
fever	1 (3.2%)	1 (4%)	0 (0%)	0.618
chest pain	6 (19.4%)	4 (16%)	2 (33.3%)	0.335
hoarseness	2 (6.5%)	2 (8%)	0 (0%)	0.474
Smoking history	16 (51/6%)	14 (56%)	3 (50%)	0.791
History of alcoholism	10 (32.3%)	8 (32%)	2 (33.3%)	0.95
Family history of tumours	4 (12.9%)	3 (12%)	1 (16.7%)	0.759
History of allergy	1 (3.2%)	1 (4%)	0 (0%)	0.618
TNM stage				
I	10 (32.3%)	9 (36%)	1 (16.7%)	0.359
II	5 (16.1)	5 (20%)	0 (0%)	
III	3 (9.6%)	2 (8%)	1 (16.7%)	
IV	13 (41.9%)	9 (36%)	4 (66.7%)	
Imaging characteristics				
Intratubular type	11 (35.5%)	9 (36%)	2 (33.3%)	0.902
Space-occupying lesions	28 (90.3%)	22 (88%)	6 (100%)	0.372
Obstructive atelectasis	11 (35.5%)	10 (40%)	1 (16.7%)	0.283
Prognostic information				
Recurrence	4 (22.2%) (n=18)	2 (8%) (n=16)	2 (100%) (n=2)	0.005
Metastasis	12 (100%) (n=12)	10 (32.3%) (n=16)	2 (100%) (n=2)	0.383
Death	9 (29%)	4 (16%)	5 (83.3%)	0.001

Red text means the value is significant.

### PMEC tumour characteristics

Regarding the sites of PMEC, the tumour occurred in either lobe of the lungs; 19/31 patients had tumours in the right lung (including the upper lobe of the right lung, the middle lobe of the right lung, and the lower lobe of the right lung), 12/31 patients had tumours in the left lung (including the upper lobe of the left lung and the lower lobe of the left lung), and 1 patient had tumours that occurred simultaneously in the middle lobe of the right lung and the lower lobe of the right lung. Bronchial obstruction or atelectasis was seen in 10/31 patients. The imaging data of all patients are detailed in **Table 1**.

### TNM staging

According to the above imaging features and tumour involvement range, staging was performed. Based on the eighth edition of the Union for International Cancer Control (UICC) criteria for TNM staging of lung cancer in 2009, of the 31 included patients, 10 had stage I disease (5 with stage 1a and 5 with stage 1b), 5 had stage II disease (1 with stage 2a and 4 with stage 2b), 3 had stage III disease (1 with stage 3a and 2 with stage 3b), and 13 had stage IV disease (10 with stage 4a and 3 with stage 4b). The pathological stage and prognosis data of all patients are detailed in **Table 2**.

**Table 2:** Univariate survival analysis of patients with pulmonary mucoepidermoid carcinoma.

	*P* value	HR	95% CI	
Sex (male/female)	0.981	0.983	0.241	4.014
Location				
upper left lung	0.879	0.897	0.222	3.62
lower left lung	0.388	0.035	0	70.149
upper right lung	0.078	3.572	0.868	14.703
lower right lung	0.281	0.318	0.04	2.548
middle right lung	0.0798	1.329	0.149	11.828
Symptoms				
cough	0.661	0.731	0.18	2.965
bloody sputum	0.783	1.215	0.303	4.871
haemoptysis	0.647	1.445	0.3	6.969
chest tightness	0.624	1.484	0.306	7.207
chest pain	0.569	1.583	0.326	7.673
Smoking history	0.407	1.771	0.459	6.84
History of alcoholism	0.022	5.707	1.281	25.412
TNM stage	0.035	5.477	1.125	26.664

Red text means the value is significant.

### Pathological examination

#### Gross examination

Among the 31 samples submitted for examination, there were 3 biopsies and 28 completely resected specimens. The maximum diameter of the tumour was 1~4 cm, with an average of 2.03 cm. The tumours were located in the larger bronchus above the segment or near the hilar bronchus. Most of them had no capsule, and the shape was irregular. They infiltrated the bronchial wall or surrounding lung tissue. The section was greyish yellow or greyish white, hard, and mainly solid, and a small amount of mucus could be seen. Intrabronchial polypoid protrusions could be seen when the tumour was located in the bronchus.

#### Microscopic examination

**Figure 1:**
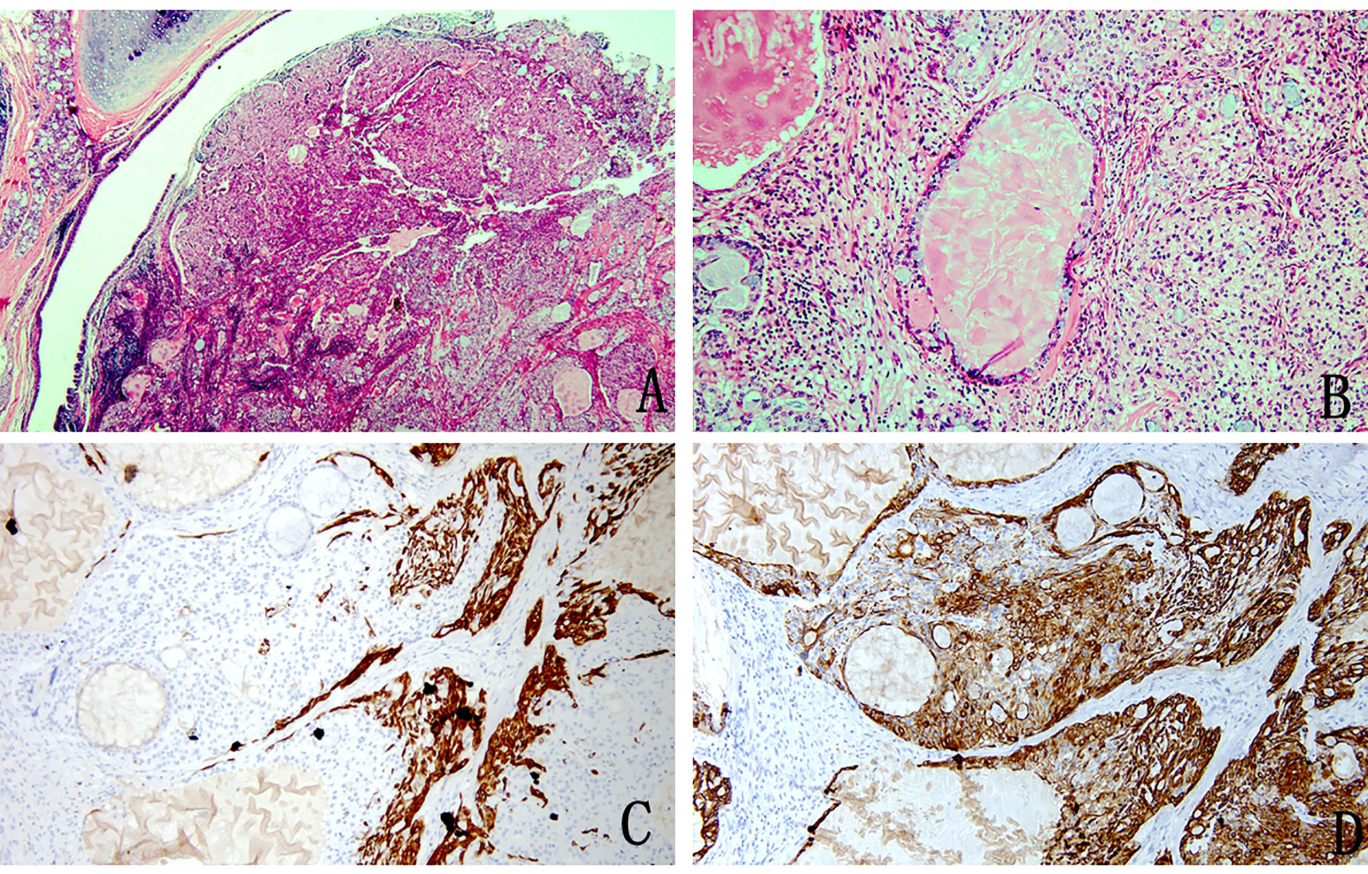
Pathology image of low-grade mucoepidermoid carcinoma **(A)** The nests of tumour cells were evident in the bronchial lumen (H&E stain, magnification × 20). **(B)** Mucus-rich columnar cells form adenoid structures with a lumen filled with mucus surrounded by nonkeratinized squamous cells and ovoid intermediate cells with eosinophilic cytoplasm (H&E stain, magnification ×200). **(C)** Immunohistochemistry showed CK5/6 squamous cell positivity, intermediate cells and columnar cell negativity (magnification × 100). **(D)** Immunohistochemical staining for CK7 was positive (magnification × 100).

**Figure 2:**
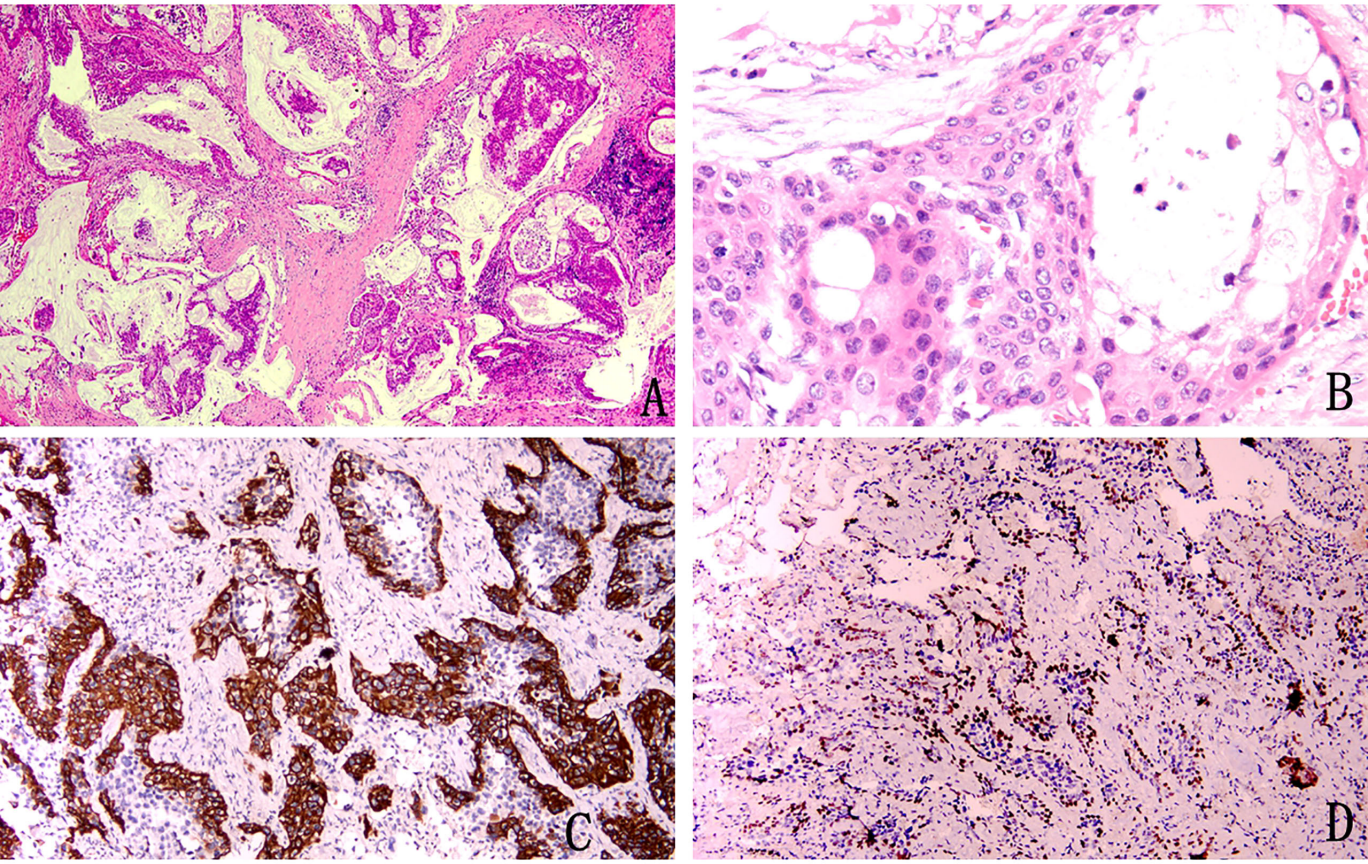
Pathological image of high-grade mucoepidermoid carcinoma **(A)** Squamous epithelium and mucus-rich columnar epithelium can be seen at low magnification (H&E stain, magnification × 40). **(B)** Squamous cells distributed in patches, columnar cells with mucus inside and outside the cells, and intermediate cells with eosinophilic cytoplasm (H&E stain, magnification × 400). **(C)** Immunohistochemical staining for CK5/6 was positive (magnification × 100). **(D)** Immunohistochemical staining was partially positive for P40 (magnification × 100).

The tumour had no capsule and showed invasive growth. The tumour was composed mainly of three kinds of cells, namely, epidermoid cells, intermediate cells and mucus cells. The three kinds of cells were mixed to form solid and glandular cystic structures. By observing the pathological morphology under a light microscope, 31 tumours were divided into 25 low-grade tumours (**Figure 1**) and 6 high-grade tumours (**Figure 2**).

### Imaging characteristics

**Figure 3:**
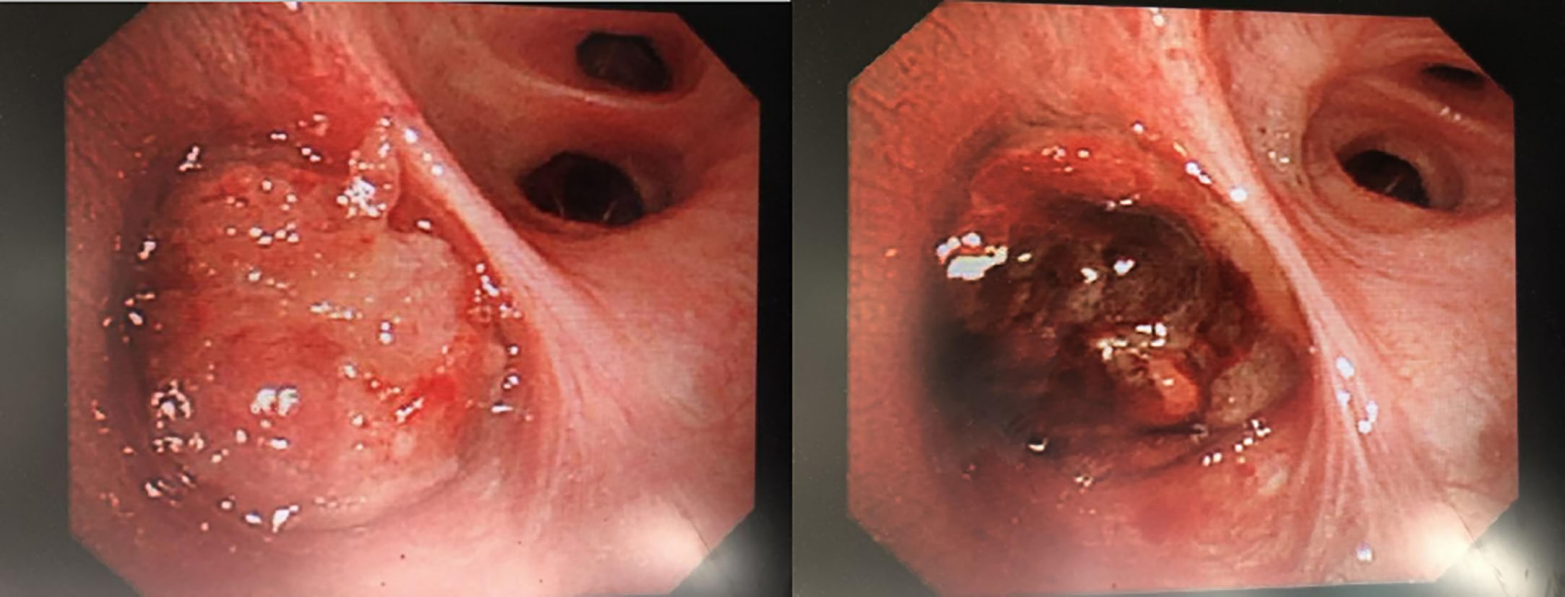
Mucoepidermoid carcinoma seen under fibreoptic bronchoscopy. Rigid bronchoscopy + electronic tracheoscopy: irregular new organisms can be seen at the opening of the right middle lobe, bad and dead objects and purplish red blood vessel attachment can be seen on the surface, which easily bleeds and blocks the orifice, and the distal end cannot be penetrated. More than 10 new bioelectric trap biopsies and frozen biopsies in the right middle lobe were sent for pathological biopsy. After the operation, the original right middle lobe was blocked, and the opening was partially unobstructed. The volume of intraoperative local bleeding was approximately 50 ml. No active bleeding was found after endoscopic haemostasis.

**Figure 4:**
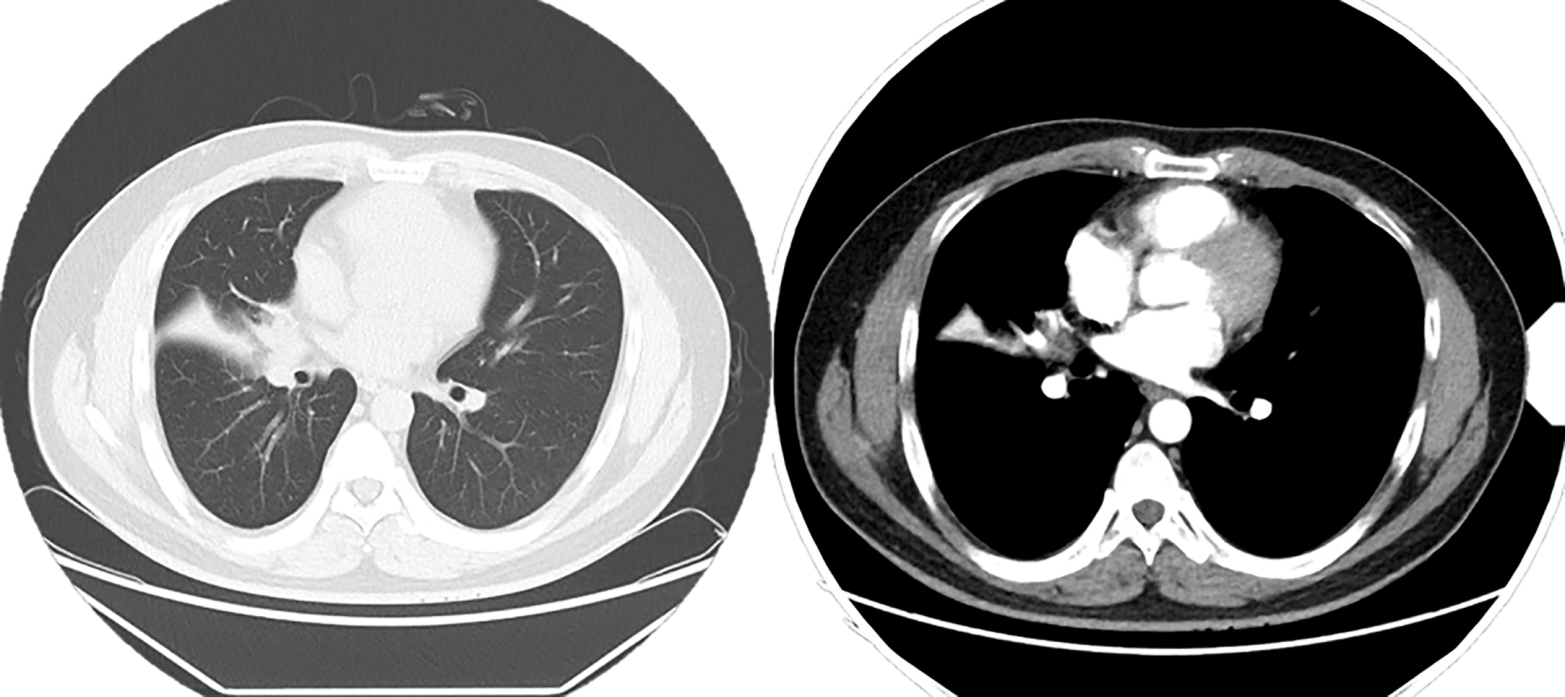
Chest CT enhancement scan: Soft tissue nodule is seen at the right hilum, measuring approximately 2.0 × 1.7 cm, and mild enhancement is seen on the right lung middle lobe atelectasis, with a patchy hyperdense shadow and right lung middle lobe bronchus occlusion. A ground-glass nodule shadow is seen in the dorsal segment of the lower lobe of the right lung, measuring 7 × 5 mm, approximately anteriorly. An enlarged lymph node shadow is seen in the right hilum, approximately 1.0 cm in diameter.

Among the 31 patients with a pathological diagnosis of PMEC, 14 underwent fibreoptic bronchoscopy, of whom 10 showed intrabronchial space occupation (**Figure 3**). All patients underwent CT, and there were 28 cases of pulmonary space occupation and 11 cases of pulmonary space occupation with obstructive pneumonia (**Figure 4**).

### Subgroup analyses

**Figure 5:**
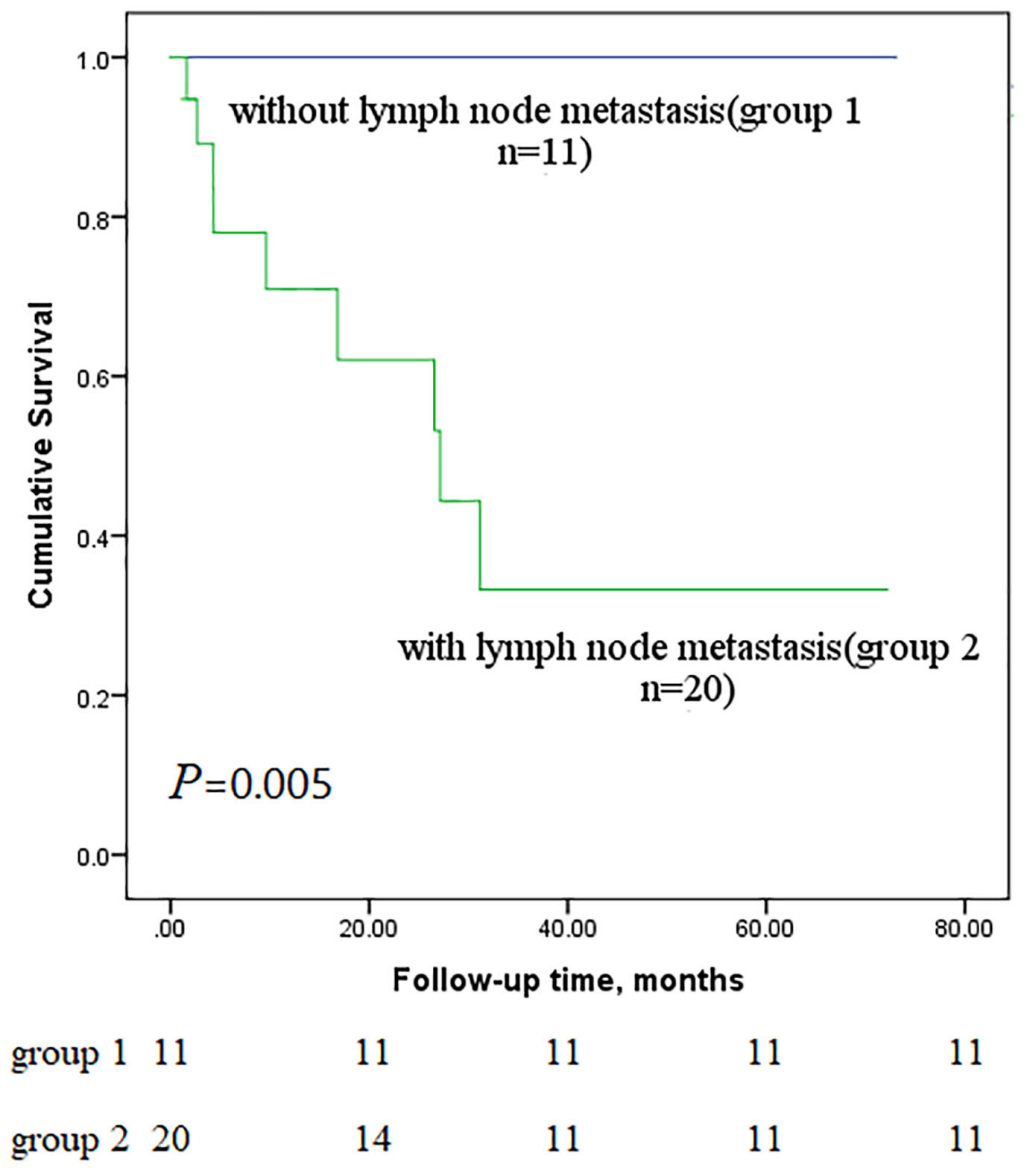
Comparison of primary outcomes between mucoepidermoid carcinoma patients with and without lymph node metastasis.

**Figure 6:**
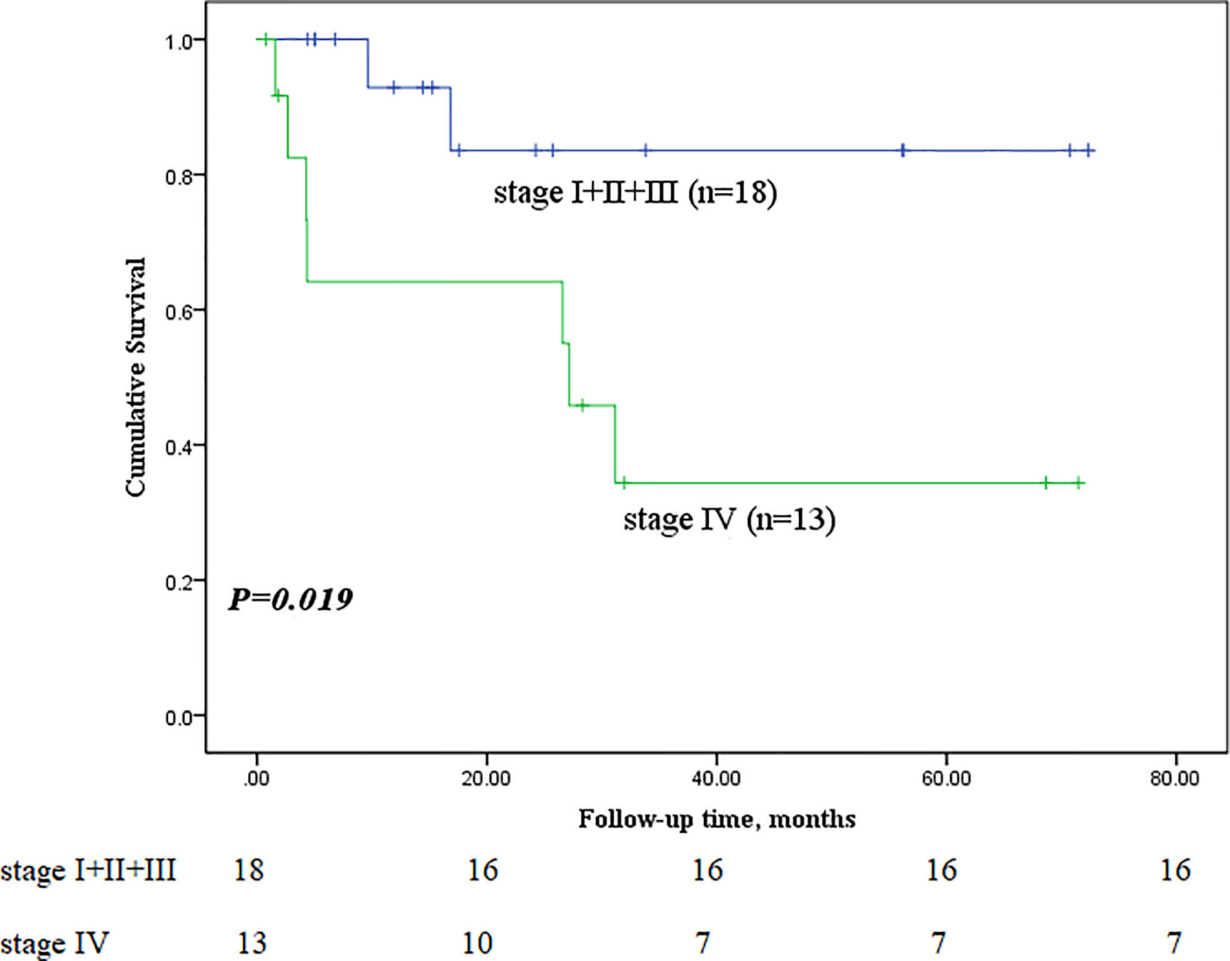
Comparison of primary outcomes between mucoepidermoid carcinoma patients with and without stage IV disease.

**Figure 7:**
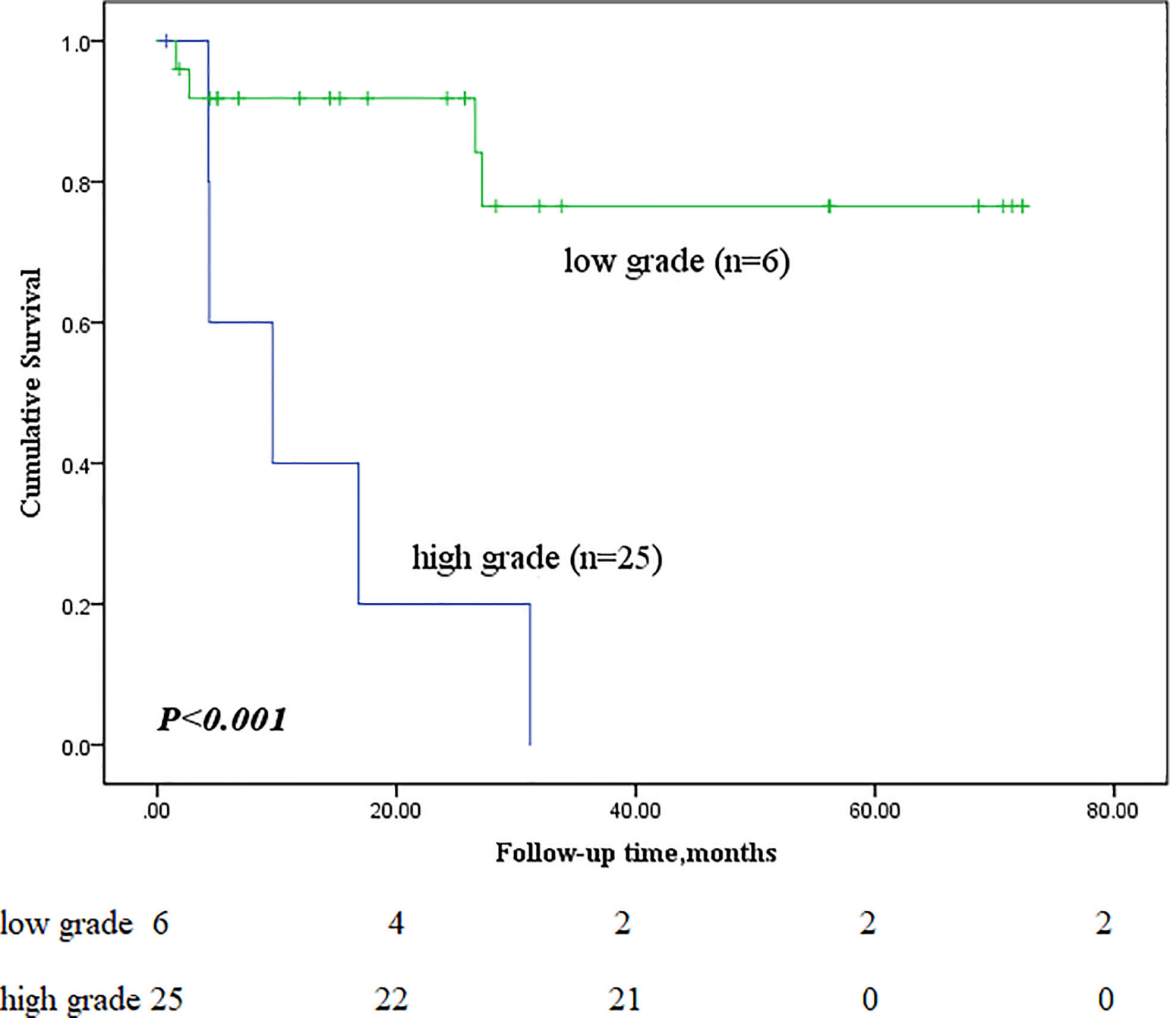
Comparison of primary outcomes between mucoepidermoid carcinoma patients with low-grade and high-grade tumours.

Next, we performed subgroup analyses to further compare outcomes between MEC patients without lymph node metastasis and those with lymph node metastasis, with and without stage IV disease, and with low-grade and high-grade MEC. None of the patients without lymph node metastasis died, whereas nine of the sixteen patients with lymph node metastasis reached the endpoint (*P*<0.001, **Figure 5**). In the survival analysis based on the presence of stage IV disease, the stage IV group had a significantly worse outcome than the nonstage IV group (𝑃= 0.019, **Figure 6**), in which 7 of 13 patients with stage IV disease and 2 of 18 patients without stage IV disease reached the endpoint. Moreover, five of six patients in the high-grade group and 4 of 25 patients in the low-grade group reached the endpoint (*P*<0.001, **Figure 7**).

### Risk factors associated with survival

Univariate Cox survival analysis of patients with PMEC showed that TNM stage IV, degree of differentiation and lymph node metastasis were risk factors for PMEC and that degree of differentiation was an independent risk factor affecting poor prognosis (*P*=0.031, HR 4.638 95% CI 0.001-0.711) (see **Table 3** for details).

**Table 3:** Multivariate Cox regression analysis of patients with pulmonary mucoepidermoid carcinoma.

Influencing factor	Index (B)	Standard error (SE)	Wald chi-squared value	*P* value	95% CI	
Age	-0.055	0.031	3.25	0.071	0.891	1.005
Sex	-1.754	1.163	2.274	0.132	0.018	1.692
Tissue differentiation level	-3.797	1.763	4.638	0.031	0.001	0.711
Lymph node metastasis	12.633	171.795	0.005	0.941	0	5234
III+IV stage	1.732	1.41	1.51	0.219	0.357	89.52

## Discussion

MEC is dominant in salivary glands and rarely occurs in the lung. Primary PMEC is a type of NSCLC, but the prognostic factors remain controversial. This investigation aimed to review cases of PMEC, analyse the prognostic factor of this disease.

The age at onset ranges from 3 to 78 years, and this disease is more often seen in adults (14). PMEC was first reported in 1952 by Smetana et al (15) and is considered less aggressive than other types of lung adenocarcinoma. In this study, after summarizing the clinical features of 31 patients with PMEC, we found that the chief complaints and imaging findings of patients with PMEC were nonspecific, which was similar to the results reported in a previous study (16). The preoperative diagnosis rate of fibrobronchoscopic biopsy was low, and the diagnosis of the patients was determined mainly by pathologic diagnosis after surgery.

In this study, 31 patients had positive findings on preoperative CT, mostly in lung space-occupying areas, partly with atelectasis or obstructive pneumonia. This result was similar to the results reported in a previous study. In Yousem’s analysis of eight cases of PMEC, in which all patients underwent CT, a single nodule or mass was found in 71% of the patients, 28% had lung consolidation, and 1% had no significant abnormality (13). In Kim’s study (17), 33% of patients had obstructive pneumonia or atelectasis on imaging. In our patients, the tumour was centrally located with smooth margins, was ovoid or lobulated in shape, was homogeneous in density and was partly calcified or mildly enhancing. Ishizumi et al (18) analysed high-resolution CT images of five PMEC and found that the lesions showed round, ovoid, or lobulated occupying spaces with smooth margins. Heterogeneous enhancement in the occupied space was demonstrated on contrast-enhanced CT, and the vascular rich sites of enhancement were confirmed by immunohistochemistry as mucus-secreting areas. Although the vast majority of PMECs have positive findings on CT imaging, CT has a limited ability to visualize the bronchial lumen, as most tumours with intraluminal lesions can have the above manifestations. A sufficiently reliable diagnosis cannot be made based on imaging data alone. Most PMECs grow into the bronchial lumen, and a fibroscopic examination allows the surgeon to make a more reliable judgement of the condition.

In this study, airway space occupation was visible in 32.3% (10/31) of patients under a fibreoptic scope, but the positive rate of pathological diagnosis was only 42.9% (6/14). Pandya et al. considered that PMEC is mostly polypoid and that the surface covers normal respiratory epithelium; thus, the positive rate of the lavage and brush test was very low, and the diseased tissue may not have been obtained if there was too little biopsy tissue captured by a bronchoscope (19). Additionally, some scholars believe that preoperative biopsy should not be performed because of the more brittle tumour texture and tendency to bleed. Therefore, the decision on whether or not to perform fibroscopy should be made according to the patient’s basic condition, tumour location, and hospital technical conditions, among other factors.

The biological behaviour of PMEC is thought to be related to the degree of differentiation, with well-differentiated tumours showing benign behaviour and vice versa. Another study compared the postoperative survival of patients with poorly differentiated NSCLC to that of patients with intermediate/well-differentiated NSCLC and found that the two were not significantly different (HR = 1.15, 95% CI: 0.81-1.64, *P* = 0.42). In this study, according to the Kaplan–Meier survival curves and Cox regression analysis, patients with low-grade bronchial MEC had better overall survival and progression-free survival times than those with high-grade PMEC. Yousem (20) suggested that advanced age and hilar lymph node metastasis predicted a worse prognosis. Vadasz’s (2) case series reported that only patients with high-grade tumours had lymph node metastasis. Univariate Cox regression analysis of the present study confirmed that age and lymph node metastasis were indeed associated with overall survival time, while it was found that TNM stage was also associated with survival. However, in the multivariate Cox regression analysis, only lymph node metastasis was an independent prognostic factor. We suggest that lymph node metastasis is the most important prognostic factor for bronchial MEC. To date, surgery remains the treatment of choice for PMEC, and the surgical approach is determined mainly by the tumour location. Twenty-one of the thirty-one patients had tumours located in the lobar bronchus, and lobectomy was the most common surgical approach and was performed in 61.9% (13/21) of all patients. If the main bronchus is invaded, then sleeve lobectomy should be considered; if it is not curative, total lung resection should be considered.

In Zheng et al’s study (7), the five-year survival rate was 100%, and all patients who died were those with stage III or IV disease. They reported five-year survival rates of only 28.6% among patients with high-grade tumours and 81.25% among patients with low-grade tumours. Kang et al (21) reported a 31% five-year survival rate for high-grade PMECs, an 80% five-year survival rate for low-grade PMECs, and an up to 80% recurrence rate for high-grade PMECs.

Some scholars believe that chemotherapy or radiotherapy is not effective for the treatment of PMEC (3, 22). Although there are reports that paclitaxel-based chemotherapeutics are considered to be effective for MEC of the salivary gland (23), reports on the efficacy of paclitaxel for the treatment of PMEC are still lacking. Therefore, some doctors do not maintain postoperative adjuvant chemotherapy or radiotherapy IV. This study shows that lymph node metastasis is an important prognostic factor, and patients with PMEC without lymph node metastasis can be treated with surgery alone. However, for patients with PMEC with lymph node metastasis, there is a certain problem with surgical treatment alone: bronchial MEC is a subtype of NSCLC, and according to the code of diagnosis and treatment for NSCLC, patients with lymph node metastasis should receive postoperative adjuvant chemotherapy. Although patients with PMEC with lymph node metastasis have a very poor prognosis and require adjuvant therapy, none of the current follow-up therapies have been indicated to be effective.

The limitations of this study are as follows. First, the patients came from the northern area of the Chinese Plain. Different geographical environments and altitudes may have disease characteristics and nutritional gene statuses that affect the mortality rate of this study. Second, the limitations of retrospective research may lead to missing or incomplete data records and offsets. Third, not all patients had characteristic MECT1 and MAML2 fusion gene detection results.

Overall, this study confirmed that for PMEC, a rare neoplasm, the clinical presentation and imaging findings are nonspecific, making it difficult to obtain an accurate preoperative diagnosis. However, we found that lymph node metastasis is an independent prognostic factor for PMEC, which can both help diagnose such patients and predict their prognosis.

## Conclusion

The preoperative diagnosis of PMEC relies on CT and bronchoscopic examination. Because the clinical manifestations are atypical, it is difficult to obtain a definite diagnosis before surgery. The survival of patients with low-grade tumours is better than that of patients with high-grade tumours. Age, grade, lymph node metastasis and TNM stage are associated with survival, and lymph node metastasis is a prognostic factor.

## Data availability statement

The raw data supporting the conclusions of this article will be made available by the authors, without undue reservation.

## Ethics statement

The studies involving human participants were reviewed and approved by No. IRB00006761-M2022102 Peking University Third Hospital. The patients/participants provided their written informed consent to participate in this study. Written informed consent was obtained from the individual(s) for the publication of any potentially identifiable images or data included in this article.

## Author contributions

All authors made substantial contributions to the conception and design of the study; the acquisition of data, or analysis and interpretation of the data; and the drafting of the article or its critical revision for important intellectual content. All authors agreed to submit the article to the current journal, gave final approval for the version to be published, and agreed to be accountable for all aspects of the work.

## Funding

This work was supported by the National Natural Science Foundation of China (No. 81900641) and the Fundamental Research Funds for the Central University: the Research Funding of PKU (BMU2021MX020, BMU2022MX008) and the Science Foundation of Peking University Cancer Hospital (grant number 2021-23).

## Acknowledgment

We thank Yanling Ding and Yongchang Sun for their contributions to the article.

## Conflict of interest

The authors declare that the research was conducted in the absence of any commercial or financial relationships that could be construed as a potential conflict of interest.

## Publisher’s note

All claims expressed in this article are solely those of the authors and do not necessarily represent those of their affiliated organizations, or those of the publisher, the editors and the reviewers. Any product that may be evaluated in this article, or claim that may be made by its manufacturer, is not guaranteed or endorsed by the publisher.
